# Balancing risk in ophthalmic prescribing: assessing the safety of anti-VEGF therapies and the risks associated with unlicensed medicines

**DOI:** 10.1007/s00417-012-2123-4

**Published:** 2012-08-12

**Authors:** Peter K. Kaiser, Alan F. Cruess, Peter Bogaert, Kamlesh Khunti, Simon P. Kelly

**Affiliations:** 1 Cole Eye Institute, Cleveland Clinic, 9500 Euclid Avenue, Desk i32, Cleveland, OH 44195 USA; 2 Department of Ophthalmology and Visual Sciences, Dalhousie University, Nova Scotia, Canada; 3 Covington & Burling LLP, Brussels, Belgium; 4 Department of Health Sciences, University of Leicester, Leicester, UK; 5 Royal Bolton Hospital NHS Foundation Trust, Bolton, UK

**Keywords:** Medication safety, Off-label prescribing, Ranibizumab, Bevacizumab, Age-related macular degeneration

## Abstract

Vascular endothelial growth factor (VEGF) inhibitor medications such as ranibizumab, pegaptanib and bevacizumab are in use for the treatment of neovascular age-related macular degeneration (AMD) and other retinal conditions, although only ranibizumab and pegaptanib are approved for these conditions. In contrast, bevacizumab was developed for the intravenous systemic treatment of colorectal cancer and is not formulated for intravitreal use, but is commonly used off-label in ophthalmology. European Union legislation permits the use of drugs outside the terms of their licence (‘off-label’) only under certain circumstances, such as during clinical trials, compassionate/named patient use in the absence of a licensed alternative, emergency scenarios (e.g., pandemics) or at the discretion of a treating physician. In such cases, patients should be fully informed regarding their treatment and any potential risks involved. Off-label drug use can be an important tool to provide patients with treatment in cases of unmet medical need. However, the use of an unlicensed medicinal product, when a suitable licensed alternative is available, puts prescribing physicians at risk of liability if safety issues arise. Emerging clinical evidence suggests safety differences exist between ranibizumab and bevacizumab.

## Introduction

Age-related macular degeneration (AMD) is the leading cause of legal blindness in the elderly [1–3]. Vascular endothelial growth factor A (VEGF-A) plays an important role in vascular homeostasis, promotion of endothelial cell proliferation and the growth of new blood vessels [4, 5]. However, expression of VEGF-A in the retina is a major mediator of angiogenesis and vascular leakage in neovascular AMD, which, if left untreated, can result in loss of central vision [6].

Intravitreal injection of a VEGF-A inhibitor is currently the primary treatment for neovascular AMD. At present, four anti-VEGF therapies are used for such patients: pegaptanib (Macugen®, Pfizer, New York City, NY, USA), alfibercept (Eylea®, Bayer HealthCare, Berlin, Germany/Regeneron Pharmaceuticals, Inc., Tarrytown, NY, USA), ranibizumab (Lucentis®, Novartis, Basel, Switzerland/Genentech, South San Francisco, CA, USA/Roche, Basel, Switzerland) and bevacizumab (Avastin®, Genentech/Roche). Pegaptanib, a ribonucleic acid aptamer that only blocks the pathologic VEGF-A 165 isoform, was the first anti-VEGF therapy to be approved for intravitreal treatment [7], although it is in limited use, as pan-VEGF-A blockers produce better outcomes [8]. Aflibercept is a fusion protein originally developed for oncology use that binds all forms of VEGF-A, as well as VEGF-B and placental growth factor (PIGF), additional angiogenic growth factors that appear to play a role in tumor angiogenesis and inflammation [9, 10]. Ranibizumab is a recombinant, affinity-matured, humanised antibody fragment (Fab) against VEGF-A produced in an *E. coli* expression system [6]. Ranibizumab was designed specifically for intravitreal use, and, in addition to AMD, is approved for the treatment of diabetic macular oedema in the European Union (EU) and macular oedema secondary to retinal vein occlusion in the EU and the USA [6, 11, 12]. Bevacizumab is a full-length, recombinant, humanised antibody to VEGF-A produced in a Chinese hamster ovary mammalian expression system [5]. As such, bevacizumab (unlike ranibizumab) is glycosylated, which prolongs systemic half-life, and contains the fragment crystallisable region (Fc region) of the antibody, which facilitates systemic absorption [13]. Bevacizumab was designed to have a long systemic half-life, important for use in oncology, and is not approved for intravitreal use [5]. Despite this, bevacizumab is often used, off-label and unlicensed, for intravitreal treatment by ophthalmologists. This practice began and spread rapidly in the period following release of the key clinical trial results of ranibizumab but prior to its approval, when ranibizumab was not yet available. Given the huge unmet medical need and rapid loss of vision in patients with AMD, there was little other choice during that time in many health economies but to use off-label bevacizumab. Thus, bevacizumab use in ophthalmology grew rapidly and has remained widespread in several economies. Currently, there is a perception that bevacizumab and ranibizumab are identical in terms of safety and efficacy. As single vials of bevacizumab intended for intravenous use can be compounded into many small doses for intraocular use, there is also a cost difference between the two drugs that some may argue takes precedence over inequalities in the safety and efficacy between the drugs [14]. However, the process of compounding results in the creation of an unlicensed medicine [15].

Several head-to-head trials of ranibizumab and bevacizumab are ongoing (Table 1). The 12- and 24- month of the Comparison of AMD Treatment Trials (CATT) study were reported in April 2011 and April 2012, respectively [16, 17]. The Inhibition of VEGF in Age-related choroidal Neovascularisation (IVAN) study released 12-month results in May 2012 [18]. For this reason, we consider it timely to evaluate the safety profiles of ranibizumab and bevacizumab, examine the need for continuing pharmacovigilance to ensure that rare adverse events (AEs) are detected for both drugs, and consider the risks, for both patients and clinicians, associated with unlicensed prescribing. A debate-style symposium at the 2nd World Congress on Controversies in Ophthalmology in Barcelona, Spain, in March 2011, centred around a discussion of these topics, and is the basis of this review.

**Table 1:** Current head-to-head trials of ranibizumab versus bevacizumab in neovascular age-related macular degeneration

Study	Country	N (target)	Trial identifier
CATT [14]	US	1208	NCT00593450^a^
IVAN	UK	600	ISRCTN92166560^b^
GEFAL	France	600	NCT01170767^a^
VIBERA	Germany	366	NCT00559715^a^
LUCAS	Norway	420	NCT01127360^a^
BRAMD	Netherlands	320	NTR1704^c^
MANTA	Austria	320	NCT00710229^a^

^a^ClinicalTrials.gov; ^b^International Standard Randomised Controlled Trial Number Register; ^c^Nederlands Trial Register

## Patient safety and the importance of post-marketing surveillance

While serious failures in patient safety are uncommon, patient safety incidents or adverse health care events are a global concern. Many such incidents are preventable—for example, around 15 % of hospital-acquired infections are thought to be avoidable [19]. Fatal adverse drug reactions are thought to be the sixth leading cause of death in the US [20]. Over 20,000 people/year in the UK are reported to have experienced serious adverse reactions to drugs [21], and this may be just a small proportion of the true figure. In UK National Health Service hospitals, patient safety incidents are estimated to occur in around 10 % of admissions, and only a fraction are reported [22]. A review of patient safety incident reports submitted to the National Patient Safety Agency from across England and Wales relating to anti-VEGF use in ophthalmic care found 166 relevant reports from 2003 to June 2010, suggesting considerable under-reporting of such incidents [23]. The incidents so reported included infection and inflammation, delays in treatment, problems with medication availability, errors in medication, and errors in the patient or eye injected [23]. It is recognised that under-reporting of adverse drug reactions is significant [24].

With licensed medications, reports from post-marketing surveillance and pharmacovigilance programmes add to data from clinical trials to build a picture of the safety profile of a drug in a given indication and patient population. Since serious safety signals may not be detected during clinical trials and may only appear during post-approval marketing [25]. collection of safety data from post-marketing studies and routine clinical use is critical. One such example is rosiglitazone (Avandia®, GlaxoSmithKline, London, UK), approved for treatment of type 2 diabetes mellitus in 2000. In 2007, a black-box warning on myocardial ischaemia was added to the label owing to concerns that had arisen in independent meta-analyses of 42 short-term randomised controlled trials involving 27,847 patients, linking rosiglitazone with increased risks of myocardial infarction and death from cardiovascular causes [26–28]. Subsequently, a number of observational studies using routine clinical data (Medicare claims data) for rosiglitazone and pioglitazone, a member of the same drug class, found a significantly increased risk of arterial thromboembolic events (ATEs) with rosiglitazone versus pioglitazone [29–31], and rosiglitazone use was suspended in Europe in 2010. Importantly, the initial clinical trials of rosiglitazone were not designed to generate cardiovascular safety outcomes data, and this AE was not detected [24]. This case demonstrates the importance of rigorous post-marketing surveillance to enable detection of serious safety signals that may not be apparent pre-registration. However, pharmaceutical companies do not typically enter into official pharmacovigilance programmes for indications that are unlicensed or off-label, as such use is at the discretion of the prescriber. In addition, if a patient dies, or misses an appointment due to disability resulting from a stroke, the ophthalmologist often does not learn of these events, which then go unreported irrespective of whether a formal pharmacovigilance programme is in place. This illustrates the importance of large-scale clinical trials such as CATT, even for off-label drugs, to assess safety in a more robust manner.

## Role of the regulatory system in protecting patients and physicians

The key principal underpinning the regulations governing the production, distribution, and use of medicines is the safeguarding of public health. For this reason, EU legislation requires a Marketing Authorisation (MA) to be granted for the purposes of placing a medicinal product on the market [32, 33]. The medicines regulatory system in the UK was developed following the thalidomide tragedy, and exists to protect the public from exposure to unsafe drugs [34]. An MA is granted if the applicant can demonstrate that the drug is safe, efficacious, and of suitable quality. Medicines must not be promoted without, or outside the terms of, their MA. Furthermore, the MA provides a clear and comprehensive description (via the Summary of Product Characteristics, SmPC) of how a medicine may be used. If a drug is prescribed in a manner outside the description given in the SmPC (or ‘label’), then this use is known as off-label (used in an indication, dosage or patient group not specified in the label), unlicensed (modified in form or strength in a way that has not been assessed or approved, such as splitting a vial into syringes) or potentially both off-label and unlicensed. The use of bevacizumab to treat AMD is an example of the latter, since both the indication and the formulation are currently unapproved.

EU regulations envisage off-label/unlicensed use only under limited ‘special need’ circumstances, including authorised clinical trials, compassionate/named patient use when no other treatment is available, emergency scenarios (e.g., pandemics) or *at the discretion of a treating physician* [32, 33, 35]. Where doctors choose to prescribe under one of the exemptions above, patients must be fully informed, in accordance with their fundamental right to be informed about the treatments they receive, about the presence of any approved alternative treatments, and be able to participate in treatment decisions [36]. The concept of informed consent for off-label/unlicensed use is reflected in the European Convention of Human Rights and associated case law, as well as in national laws and ethical guidance [37]. National requirements for informed consent vary across the EU, but it is generally agreed that physicians should inform their patients of the unlicensed nature of the proposed treatment, the reasons for proposing the treatment, any potential side-effects, the risks and benefits, and available alternatives [37–42]. For example, in the UK, General Medical Council (GMC) guidance on prescribing off-label/unlicensed medicines states that:

[Y]ou must explain the reasons for prescribing a medicine that is unlicensed or being used outside the scope of its licence where there is little research or other evidence of current practice to support its use, or the use of the medicine is innovative [43, 44].

While the use of a drug outside the terms of its licence can be an important tool to provide patients with treatment in cases of unmet medical need where there are no licensed therapy options, the use of an unlicensed medicinal product, when a suitable tested and approved alternative is available, may put prescribing physicians at risk of liability if safety issues arise. Should any untoward event arise through the use of that drug, the treating physician would have the burden of proof to demonstrate that its use was performed as standard of care [45]. By prescribing licensed drugs, physicians can be confident that the risk–benefit balance of the therapy has been assessed and is supported by quality pre-clinical and clinical data. Physicians should also bear in mind that cost is not a relevant consideration when deciding whether to prescribe a drug off-label. In the UK, GMC guidance does not allow doctors to factor in the cost of a medicine [43, 44]. Rather, doctors must be satisfied that an unlicensed or off-label medication would better serve the patient’s needs than an appropriately licensed alternative, and be satisfied that there is a sufficient evidence base and/or experience of using the medicine to demonstrate its safety and efficacy. In March 2012, the Court of Justice of the European Union ruled that a Polish law allowing the import and sale of unapproved and less costly medications to similar, approved drugs under the ‘special need’ exemption breaches EU law [46]. The ruling noted that the exemption must be used only when completely essential and based solely on therapeutic need. If approved medicines with the same active ingredients, dosages, and forms are on the market, there cannot be a requirement for ‘special needs’, and financial considerations cannot be used as a justification.

## Safety of anti-VEGF therapies in the treatment of neovascular AMD

As a result of the important role of VEGF in promoting vascular homeostasis, there is a theoretical risk of ATEs following the use of any VEGF inhibitor [4, 47, 48]. When anti-VEGF agents are given systemically, there is a known risk of increased blood pressure and ischaemic cardiac events. This is noted in the black-box label of bevacizumab. This is important, since studies have shown increased risks of stroke and coronary heart disease in untreated patients with AMD compared with controls [49–51]. A difference in the rates of these events in patients with AMD treated with ranibizumab and bevacizumab would be of particular clinical relevance.

As bevacizumab and ranibizumab are derived from the same mouse monoclonal anti-VEGF antibody, they are sometimes perceived to have equivalent safety and efficacy [14]. However, as reviewed recently [52], bevacizumab and ranibizumab are different at the molecular level, so safety and efficacy data cannot be extrapolated. While bevacizumab is a full-length recombinant antibody, ranibizumab was developed by inserting sequences from the parent antibody into a human antigen binding fragment (Fab) framework. The humanised Fab was then selectively mutated by changing six amino acids to increase its affinity for binding and inhibiting VEGF-A over the original mouse antibody and bevacizumab [6]. As an antibody fragment, ranibizumab has a relatively short systemic elimination half-life, estimated at ~2 hours compared with ~20 days for bevacizumab, and lacks the Fc region of an antibody (present in bevacizumab), which has been reported to initiate complement activation and an immune response [53, 54]. In addition, bevacizumab reaches the systemic circulation following intravitreal injection more rapidly than might be expected for a full-length antibody. Experiments in rabbits demonstrated the vitreous half-life of intravitreal bevacizumab to be 4.3 days, similar to that of the much smaller molecule of ranibizumab (2.8 days) [55, 56]. An explanation for this has been provided by studies in mice indicating that the neonatal Fc receptor, expressed by retinal pigment epithelial cells, is involved in the active transport of bevacizumab across the blood–retina barrier [13]. This receptor may be upregulated in eyes with neovascular AMD, leading to rapid elimination of bevacizumab from the vitreous humor into the systemic circulation [13]. In comparison, a study of the half-life in humans measured intraocular concentrations of bevacizumab from 29 patients, and found the half-life of bevacizumab to be dose-dependent. The half-life of bevacizumab from a single 1.5 mg dose was determined as 7.85 days, compared with 11.67 days for a 3.0 mg dose [57]. Studies in rabbits detected bevacizumab, but not ranibizumab, in the serum following intravitreal injection, suggesting that bevacizumab may be more likely to have clinically observable systemic effects than ranibizumab [55, 56]. Importantly, systemic VEGF inhibition has been demonstrated in patients with diabetic retinopathy following intravitreal bevacizumab injection [58]. Plasma VEGF levels were significantly reduced for at least 30 days (*p* < 0.001) after a single bevacizumab injection [58]. Similarly, patients with AMD treated with intravitreal bevacizumab have a significant reduction in plasma VEGF levels up to 28 days after injection (*p* < 0.001), but no significant reduction in plasma VEGF following ranibizumab treatment [59].

The molecular and pharmacological differences between ranibizumab and bevacizumab signal that safety and efficacy data from one cannot be extrapolated to the other. An accumulating body of clinical and regulatory evidence supports the hypothesis that these fundamental differences translate into clinical differences in the safety profile of these two drugs.

**Fig. 1:**
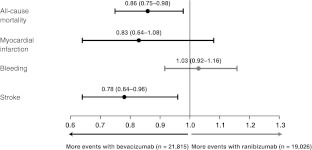
Hazard ratios adjusted for patient characteristics (with 95 % confidence intervals) for systemic adverse events at 1 year in the secondary analysis of a Medicare claims database study (*n* = 40,841) [61]

A recent, non-randomised, comparative study in patients with AMD confirmed bevacizumab as an independent and significant predictor for new ATEs, associated with around a 10-fold higher risk than ranibizumab [60]. Two independent retrospective analyses of the US Medicare claims database also report differences in rates of systemic AEs with ranibizumab and bevacizumab [61, 62]. The first examined data from 146,942 patients with AMD, to compare the risks of several systemic AEs with current treatments for AMD. While the primary analysis of this study did not identify significant differences in risk between the ranibizumab and bevacizumab groups, a secondary analysis in which the study population was limited to 40,841 newly-treated patients who received bevacizumab or ranibizumab only showed significantly increased risks of all-cause mortality and stroke with bevacizumab (Fig. 1) [61]. These results are consistent with those of a second (unpublished) Medicare data analysis, which showed a significantly increased risk of mortality with bevacizumab versus ranibizumab in an analysis of 77,886 claimants [62]. While the limitations of such database analyses are recognised, neither showed fewer systemic ATEs with bevacizumab, indicating that there may be an emerging safety signal, as was the case with the early warning signs with rosiglitazone discussed above.

Several prospective comparative studies of ranibizumab and bevacizumab are ongoing. None have the statistical power to determine if a safety difference exists between the drugs. Considering the CATT study primary efficacy endpoints at 12 months, monthly injections of bevacizumab successfully demonstrated non-inferiority to monthly ranibizumab as well as ranibizumab given as needed [16]. It is important to note that in Year 1 there were no differences in venous thrombolic events or ATEs; however, there was a significantly higher rate of serious systemic AEs (of which 80.5 % were associated with hospitalisation) with bevacizumab compared with ranibizumab, which remained significantly higher after adjustment for baseline demographics and coexisting illnesses (*p* = 0.04) [14]. In CATT Year 2, ranibizumab maintained visual acuity gains from Year 1 with both regimens [17]. Greater numerical visual acuity gains and significantly fewer injections were seen with ranibizumab versus bevacizumab as needed, and the 2-year CATT data confirmed an overall significantly higher risk of serious systemic adverse events with bevacizumab versus ranibizumab (*p* = 0.004) [17]. ATEs, systemic haemorrhage, congestive heart failure, venous thrombotic events, hypertension, and vascular death were more frequent in bevacizumab-treated patients, and as in Year 1, there were significantly more gastrointestinal disorders in patients treated with bevacizumab compared with ranibizumab (*p* = 0.005) [17].

Similarly to CATT, the 2-year IVAN study includes comparisons between ranibizumab and bevacizumab and between regimens [18]. However, there is no comparison between individual treatment arms, due to the study lacking the statistical power to compare these groups. The primary endpoint of the study was corrected visual acuity after 2 years. The Year 1interim analysis showed that improvement in visual acuity was greater in the ranibizumab arms than in the bevacizumab arms [18]. Bevacizumab failed to demonstrate non-inferior vision gains to ranibizumab at 1 year using a 3.5 letter non-inferiority margin (95 % CI, −4.04, 0.06; *p* = 0.056) [18]. Secondary findings (e.g., fluid on optical coherence tomography, dye leakage on angiogram) also favoured ranibizumab [18]. There was no significant difference in the proportion of patients experiencing at least one serious adverse event (SAE) with ranibizumab versus bevacizumab in IVAN at 1 year [18]. However, there were numerically more SAEs with bevacizumab (*n* = 37) than with ranibizumab (*n* = 30) [18].

Given the relatively small study populations in CATT, IVAN, and the other current head-to-head trials, it is unlikely that any of these studies are powered to reveal significant differences in rates of individual serious AEs between treatment groups [63]. There is the possibility that a meta-analysis of these trials may reveal a safety signal, and there remains a huge need for large studies powered for safety to elucidate fully the incidence of infrequent serious AEs.

In addition to the fundamental structural and pharmacokinetic differences between the two drugs, manufacturing and packaging specifications also differ between ranibizumab and bevacizumab. Ranibizumab is manufactured to meet United States Pharmacopeia (USP) standards for ophthalmic solutions (USP789), which are more stringent with regard to sterility and particulate matter than the requirements for intravenous medications such as bevacizumab (USP788) [64]. In the latter, more particulate matter is tolerated [65], which has the potential to cause irritation and inflammation if injected into the eye. Once manufactured, ranibizumab is packaged into sterile, single-use vials. In contrast, bevacizumab vials are intended for single use as an intravenous infusion, and in practice undergo compounding into smaller aliquots for use in AMD care [66]. Active immunoglobulin levels in compounded bevacizumab samples have been shown to vary significantly between compounding pharmacies, leading to the possibility of variable efficacy depending on the amount of antibody present [67]. In addition, compounded bevacizumab samples have also been shown to have an increase in particulate matter, which may lead to elevated intraocular pressure and inflammation [67].

The recent analysis of Medicare claims data reported that patients given bevacizumab were significantly more likely to require treatment for ocular inflammation than those receiving ranibizumab [62], and a precautionary recall of compounded bevacizumab was issued in March 2012 by a leading National Health Service (NHS) hospital compounding pharmacy in the UK after a number of reports of suspected sterile endophthalmitis following intravitreal injection [68]. Whenever the physician does not control the source of drugs, there is an increased potential for human error, either by an incorrect dose or incorrect drug [69]. A recent series of endophthalmitis cases in China was traced back to counterfeit bevacizumab being used by the compounding pharmacy [70]. Finally, there is an increased risk of contamination when single-use vials are accessed multiple times. Clusters of cases of endophthalmitis traced back to such contamination led to an alert being issued by the US Food and Drug Administration [71]. More recently, the Medical Products Agency in Sweden issued a position statement in March 2012 recommending the use of ranibizumab for retinal conditions, due to concerns that available safety data for bevacizumab in this setting are inadequate, but indicate an increased risk for ocular inflammation and potentially also for certain systemic adverse events [72]. In February 2012, the Emilia–Romagna region of Italy temporarily suspended the off-label use of bevacizumab for newly diagnosed patients following a response from the Italian Medicines Agency (AIFA) to a request from Emilia–Romagna to reimburse bevacizumab through a law that regulates the off-label use of drugs at national level [73]. AIFA stated that the results of the CATT study do not justify the off-label use of bevacizumab, both in terms of safety and efficacy [73]. In contrast, having previously supported and endorsed use of bevacizumab in patients [74], in 2012 the German Ophthalmological Society, the Retinological Society, and the Association of Ophthalmologists in Germany released a new statement on therapeutic strategies for treating wet AMD in response to the CATT study report, which refers to a similar efficacy of bevacizumab but also acknowledged the legal implications of off-label use [75]. In December 2011 the Royal College of Ophthalmologists stated that the College believes the use of ranibizumab for AMD should be the default position until the NHS commissions a National Institute for Health and Clinical Excellence (NICE)/ Medicines and Healthcare products Regulatory Agency (MHRA) assessment of bevacizumab and until a national policy is formulated [76]. To date, no such new national policy in the UK has been developed.

## Discussion

Debate currently surrounds the use of unlicensed treatments in medicine, with wide-reaching implications for informed consent, patient safety, and the regulatory process itself. It is our hope that greater understanding of these issues by ophthalmologists may lead to better patient care in ophthalmic practice.

The primary role of regulatory systems is to promote patient safety. Despite this, the regulatory apparatus surrounding post-marketing monitoring and safety is less coherent than that concerned with the drug discovery, development and MA process. Only in recent years, following public and media interest in high-profile drug safety cases such as those of rosiglitazone and rofecoxib (Vioxx®, Merck, Whitehouse Station, NJ, USA), is the process of proving drug safety receiving similar attention to the process of demonstrating efficacy, and this has led to reform of EU pharmacovigilance laws. However, in order to make real differences in patient safety, regulatory bodies must make rational safety decisions and allocate resources to implement changes. Policy decisions must be made considering all available evidence. In particular, patient safety must take precedence over economic considerations. Professional bodies could have an important role in raising safety concerns in ophthalmology in general and with regulatory authorities. For example, the Royal College of Ophthalmologists has provided detailed guidance on patient safety in ophthalmology, which should be of merit to eye-care teams to improve patient safety in local departments and aims to keep members updated of emerging issues [72, 77].

Ranibizumab and bevacizumab are distinctly different at the molecular level, have different target profiles and different approved routes of application. These differences mean that safety and/or efficacy data from one cannot be extrapolated to the other, and the rosiglitazone case provides a timely illustration that agents in the same drug class can have significant differences in safety profiles. Since bevacizumab is unlicensed for use in ophthalmology, post-marketing pharmacovigilance normally implemented for licensed drugs has not been carried out to date. Existing clinical evidence comes solely from clinical studies, with a number of studies reporting trends towards an increased risk of systemic AEs with bevacizumab [16–18, 57–59]. Though such studies have limitations, the consistency of findings reinforces the plausibility of patient safety differences between these VEGF inhibitor agents. However, large studies specifically powered for safety are needed to reveal the significance of rare serious AEs with these agents. The issues discussed here regarding pharmacovigilance and unprofessional compounding of bevacizumab could be addressed by professional organizational bodies introducing guidelines in these matters.

Physicians may be liable for serious AEs that occur in patients they treat, particularly if they have not provided patients with sufficient information about the unlicensed drug to allow them to provide informed consent. This leads to particular concerns for ophthalmologists who may be encouraged or even pressured to prescribe off-label bevacizumab by their employing institutions or insurance companies. In such situations, it is even more important that the patient is fully informed.
